# Supplementary material for: An Audit of the Nutrition and Health Claims on Breakfast Cereals in Supermarkets in the Illawarra Region of Australia
Source: Nutrients. 2019 Jul 15;11(7):1604. doi: 10.3390/nu11071604 (PMC6683094; doi:10.3390/nu11071604)
Supplement: Supplementary file 1 [file nutrients-11-01604-s001.pdf]

SUPPLEMENTARY MATERIAL

Table S1. Summary of key concepts specific to this research

Term	Abbreviation	Purpose	Examples relevant to this research
Food Standards Australia New Zealand	FSANZ	Independent statutory agency in the Australian Government Health portfolio, responsible for developing standards related to food	Develops the Food Standards Code (FSC).
Food Standards Code	FSC	Provide standards and policies regarding food safety, food labelling, food additives, production and processing standards etc.	Standard 1.2.7 is a component of the FSC.
Standard 1.2.7	-	Outlines regulations regarding food labelling, claims and advertising. Figure 1 lists examples of claims.	Standard 1.2.7 was used to determine claim compliance for nutrition and health claims. Nutrition claims are compliant if they meet the 'qualifying criteria', health claims are compliant if they meet the 'qualifying criteria' and the NPSC.
Nutrient Profiling Scoring Criterion	NPSC	Nutrient profiling is a means to categorise foods according to their nutritional composition. In Australia the NPSC is used to classify foods as 'healthy' or 'less healthy' as products are allocated a NPSC score according to their nutritional profile. For food products to be eligible to carry health claims, they must meet the NPSC 'healthy' criteria. Therefore, it was proposed if foods could be classified according to the NPSC (how healthy a product is) this would reduce consumer confusion regarding health claims.	The NPSC allocates 'baseline points'; energy, saturated fat, sugar and sodium, 'modifying points'; fruit, vegetables, nuts, legumes (FVNL), dietary fibre(F) and protein(P). The final score is calculated by subtracting the 'modifying points' from the 'baseline points' (baseline – modifying [(FVNL) – (F) – (P)]). Breakfast cereals are classified as 'healthy' if the NPSC score is less than 4.
Grains and Legumes Nutrition Council	GLNC	The FSC does not provide standards regarding wholegrain food claims. The GLNC regulates and administers the Voluntary Code of Practice for Whole grain Ingredient Content Claims.	This Code was used to assess compliance of wholegrain claims in this research.

Figure S1: Flow chart of excluded claims from analysis in this study

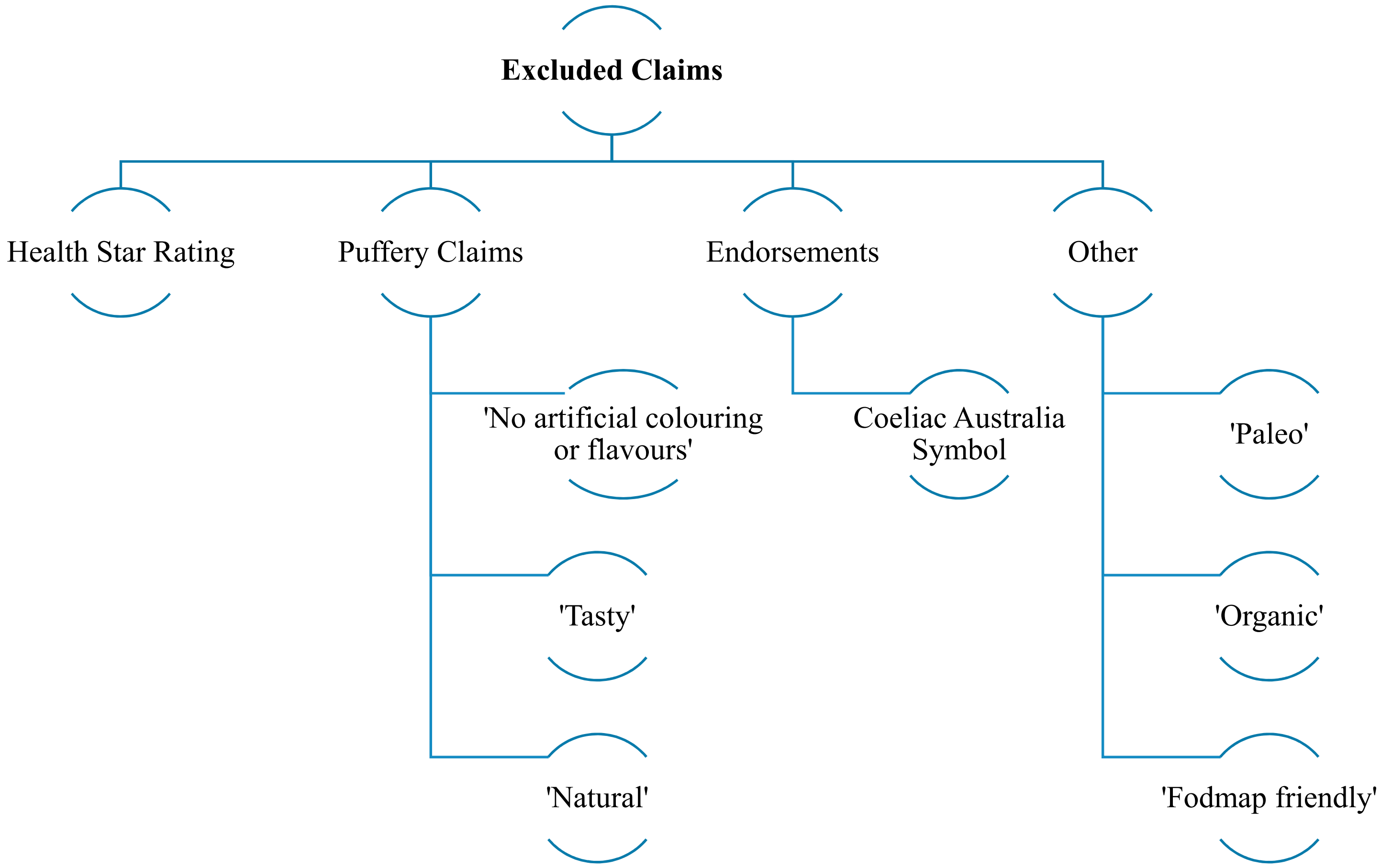

Table S2. Most commonly used nutrition and health claims found to be carried on breakfast cereal products in the supermarket audit

Claim type and subject	Number of claim (n)	Percent of all claims (%)
**Other**	337	25.7%
**Content: Source of fibre/contains fibre**	121	9.2%
**Content: Good source of fibre**	95	7.3%
**Content: Source of/contains protein**	75	5.7%
**Content: Gluten free**	46	3.5%
**General: Fibre**	36	2.8%
**Content: Low sodium**	32	2.4%
**Content: Good source of folate**	32	2.4%
**Content: Good source of thiamine (B1)**	27	2.1%
**Less common claims; contribute between 1.00 - 1.99% of all claims***	295	22.5%
**Less common claims; contribute between 0.00 - 0.99% of all claims***	213	16.3%
**Total**	**1309**	**100.0%**

*The less common claims found in this study have been grouped together for simplicity of results as presented in the last two rows.

Table S3. Breakfast cereal products surveyed in the supermarket audit that meet the FSANZ NPSC

Breakfast cereal category	YES		NO		Total
	**Number of products that meet the NPSC (n)**	**% of products per category that meet NPSC**	**Number of products that do not meet NPSC (n)**	**% of products per category that do not meet NPSC**	**(n)**
**Biscuits & bites**	28	87.5	4	12.5	32
**Brans**	37	92.5	3	7.5	40
**Bubbles, puffs, flakes**	8	38.1	13	61.9	21
**Health & wellbeing**	53	88.3	7	11.7	60
**Kids cereal**	10	50.0	10	50.0	20
**Muesli**	91	84.3	17	15.7	108
**Oats**	48	100.0	0	0.00	48
**Total**	**275**		**54**		**329**

Table S4. Most common non-compliant nutrition content claims carried on supermarket breakfast cereal products in the Illawarra region

Claim Subject	Claim Number (n)	% of all non-compliant nutrition claims
**Other**	41	58.6%
Other - whole grain	31	75.6% (of all other claims)
**Good source of riboflavin (B2)**	8	11.4%
**Gluten free**	5	7.1%
**Good source of fibre**	5	7.1%
**Less common claims contributing 0.00-4.99%**	11	15.7%
**Total**	**70**	**100.0%**
